# Successful Management of Coronary Artery Rupture with Stent-Graft: A Case Report

**DOI:** 10.1155/2014/391843

**Published:** 2014-07-13

**Authors:** Berkay Ekici, Aycan Fahri Erkan, Utku Kütük, Hasan Fehmi Töre

**Affiliations:** Department of Cardiology, Faculty of Medicine, Ufuk University, Mevlana Bulvarı (Konya Yolu) No. 86-88, Balgat, 06520 Ankara, Turkey

## Abstract

Perforation of coronary arteries is a relatively rare yet life-threatening complication of percutaneus coronary interventions and is encountered in approximately 0.5% of these procedures. According to the type of coronary perforation, various methods of correction are employed, ranging from conservative approach to emergency cardiac surgery. Coronary stent-grafts are composed of two metal stents and a polytetrafluoroethylene layer between them. Advent of such stents enabled efficient endovascular repair of coronary artery perforation. We present a case of coronary artery perforation which had occurred during stent implantation for the treatment of a bridged segment in the distal portion of the left anterior descending artery. This perforation was successfully managed by implanting a stent-graft.

## 1. Introduction

Coronary perforation (CP) is a rare percutaneous coronary intervention (PCI) complication which may lead to pericardial effusion frequently accompanied by tamponade. CP is often mortal when it is undiagnosed or untreated. Due to the expanding volume of PCI procedures worldwide, a rapid increase in the number of CP cases has been observed in the last decades. Nevertheless, definitive management of CP has not been established yet [1–3].

## 2. Case Presentation

**Figure 1:**
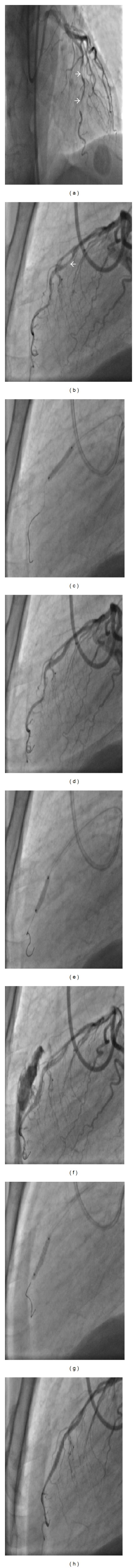
((a)-(b)) Coronary angiography images of the patient in anterior-posterior cranial and lateral views which show myocardial bridges causing severe compression and near complete occlusion of the middle and distal segments of the LAD during systole, marked by arrows. ((c)-(d)) A drug eluting stent deployment at the middle segment of the LAD in lateral view. ((e)-(f)) During stenting of the distal segment of the LAD, coronary artery perforation occurred and massive blood flow extravasating into the pericardial space. ((g)-(h)) A polytetrafluoroethylene-covered stent was deployed over the perforation site, and this successfully sealed the perforation. LAD: left anterior descending.

A 74-year-old man was admitted to the cardiology department of our institution with a history of chest pain and dyspnea on exertion. His functional capacity was class II according to the New York Heart Association (NYHA) classification. He had a history of stent implantation in the proximal left anterior descending coronary artery (LAD) two years ago, in another facility. Physical examination was unremarkable except for the hypotension (90/50 mmHg) and bradycardia (57 beats per minute). A chest X-ray revealed moderate cardiomegaly. The patient's complete blood count, biochemical analyses, and the coagulation tests were normal. An electrocardiogram (ECG) showed sinus rhythm and nonspecific ST-T segment changes. Transthoracic echocardiography images revealed hyperdynamic left ventricular systolic function, grade I diastolic dysfunction, left ventricular regional wall motion abnormality (mild anterior wall hypokinesia), and mild tricuspid and mitral regurgitation. In order to investigate the etiology of the chest pain, dobutamine stress echocardiography was performed, revealing worsening hypokinesia of the anterior wall. Due to positive dobutamine stress echocardiography and recurring angina, coronary angiography was performed. It revealed nonsignificant atherosclerotic plaques in the circumflex artery and the right coronary artery, a patent stent in the proximal LAD, and myocardial bridges causing severe compression and near complete occlusion of the middle and distal LAD during systole, with pulsatile contrast hanging or a “milking effect.” Despite medical therapy with acetylsalicylic acid, a *β*-blocker, and a calcium antagonist, the patient continued to experience dyspnea and chest pain during exercise; therefore PCI for the treatment of the bridged segments in the LAD was planned. After administration of a 600 mg loading dose of clopidogrel and an intravenous bolus of 10.000 units heparin, left main coronary artery was cannulated with a 7F JL4 guiding catheter and a 0.014′′ floppy guide wire was advanced. The middle segment of the LAD which was almost totally compressed by the myocardial bridge was stented using a 2.75 × 18 mm drug eluting stent (DES) deployed at 16 atmospheres. There was no residual stenosis, dissection, or evidence of compression by the myocardial bridge at the end of this procedure. During stenting of the bridged segment in the distal portion of the LAD with a 2.75 × 14 mm DES at 18 atmospheres, CP occurred and angiographic images showed massive and pulsatile extravasation from the LAD into the pericardial space (Figure 1). An immediate drop in blood pressure and heart rate was noted, and the patient complained of severe chest pain. Bigeminal ventricular premature complexes and slight ST segment depression in the anterior precordial leads were noted on the ECG. A stent-graft 19 mm in length connected with a 3 mm balloon was implanted immediately in the site of the rupture, with subsequent complete restitution of blood flow in the LAD and termination of extravasation into the pericardial space. The chest pain abated and the ECG was normalized. Protamine sulfate (50 mg) was administered intravenously to reverse the effect of heparin after intervention. Ibuprofen (800 mg 1 × 2 p.o.) was prescribed as an analgesic and anti-inflammatory medication. Transthoracic echocardiography in the catheter laboratory and one day after the PCI procedure showed minimal intrapericardial fluid but no progression was observed. The patient was followed up for 2 days in the coronary care unit and was discharged from the hospital 1 week later. At the follow-up visits at 30 days and 2 months, the patient's NYHA class was improved and chest pain was alleviated. The patient is currently on follow-up with medical treatment for 4 months and long-term dual antiplatelet therapy for at least 1 year has been planned.

## 3. Discussion

Coronary artery perforation is a rare but potentially fatal complication of PCI that can result in life-threatening cardiac tamponade. In the present era, the incidence of CP ranges from 0.19% to 0.59% [4–6]. CP occurs when a dissection or intimal tear completely penetrates the arterial wall leading to either vessel puncture with minimal dye staining or vessel rupture with brisk extravasations of blood and dye into the pericardial space. Several factors can be associated with these conditions:

1. clinical variables: as advanced age, female sex, renal dysfunction, and non-ST-segment elevation myocardial infarction;
2. angiographic factors: chronic total occlusion, coronary artery calcification, tortuous vessels, target lesions in the circumflex and right coronary arteries, long target lesions (>20 mm), and eccentric lesions;
3. technique-associated factors: use of hydrophilic/extra stiff wires, atherectomy devices, increased balloon to artery ratio, intravascular ultrasound guided PCI optimization and high-pressure stent postdilatation, percutaneous excimer laser coronary angioplasty, and cutting balloons [7–9].

Coronary perforation is encountered most frequently due to distal migration of guide wire and/or wire fracture or accidental positioning of the guide wire outside the course of the coronary artery. Hydrophilic-coated guide wire may increase the risk of perforation because they more easily migrate to the distal portion of the vessel [10]. The CP classification by Ellis et al. [11] has received world-wide acceptance: Type I, extraluminal crater without extravasation; Type II, pericardial or myocardial blushing; Type III, perforation ≥ 1 mm diameter with contrast streaming; and cavity spilling.

Traditional management of CP consists of prolonged balloon inflation (proximal to or at the site of perforation to prevent tamponade) and reversal of anticoagulation with protamine. It has been reported that the administration of protamine in patients with CP seems to be safe, without an increase in the risk of vessel/stent thrombosis. Platelet transfusion is recommended in patients who had received abciximab. If the renal function is normal, eptifibatide and tirofiban infusions may be terminated with rapid reversal of effect owing to their short half-lives. The development of pericardial tamponade is not affected by the use of glycoprotein (GP) IIb/IIIa antagonists. Continuation of antiplatelet therapy is not associated with overt rebleeding [7]. In a meta-analysis of three randomized trials, no significant difference was found in composite end points including complicated CP between bivalirudin monotherapy and the use of unfractionated heparin plus GP IIb/IIIa inhibitors [12]. Pericardiocentesis must be performed promptly if tamponade occurs. Surgical repair of CP comprises either ligation or suturing of the vessel and bypass grafting to the distal portion of the vessel. In addition, pericardial patch/Teflon felt wrapping repair of the CP with or without coronary bypass grafting is recommended particularly when multiple stents with CP and subepicardial hematoma are present [13].

The incidence of adverse outcomes, emergency coronary artery bypass grafting, and death significantly decreased in the present era [14]. Stent-grafts for emergency implantation in case of CP must be an obligatory inventory of catheterization laboratories. The polytetrafluoroethylene-coated stent is a safe and effective alternative which can be used for sealing a major CP and obviating cardiac surgery. The utilization of synthetic stent-grafts is less invasive, faster, and more effective when compared to surgical interventions and is generally considered to be the gold standard in the management of CP [15]. The incidence of subacute thrombosis and restenosis in the stent-grafts is relatively higher than in the conventional coronary stents (10.3% versus 3.4%, resp.), which may be related to delayed endothelialization and increased susceptibility to thrombus formation in these stents. Therefore, long-term dual antiplatelet therapy at least for 1 year is recommended [16]. In our case, the rapidly forming massive pericardial effusion and hemodynamic instability led to the decision of urgent intervention, and a stent-graft was implanted immediately. Angiographic images after the stent-graft procedure were satisfactory and the CP had been totally sealed. The rupture in this case may have occurred due to advanced age, high-pressure balloon dilatation, relatively small diameter of the vessel, and the fragility of the vessel wall between the bridged segment and the normal segment. Nevertheless, the information about CP during stenting of bridged segments is limited, and the topic warrants further research.

Stenting of a myocardial bridge is still controversial due to high restenosis rates, potential plaque prolapse, and risk of stent fracture. Myocardial bridging is asymptomatic in most of the cases, but in rare instances it may be associated with serious complications such as acute coronary syndromes, lethal arrhythmias, and even sudden death. Symptomatic patients with objective evidence of ischemia on noninvasive stress tests and which are refractory to medical therapy, PCI, or surgical treatment should be preferred [17]. Our case may be interesting due to the nature of the target lesion (a bridging segment), and to the best of our knowledge such cases were not described constantly. In our case, the patient was symptomatic despite therapy with acetylsalicylic acid, a *β*-blocker, and a calcium antagonist; therefore PCI for myocardial bridging middle and distal part of the LAD artery was performed.

## 4. Conclusion 

As a conclusion, in cases of CP, the importance of timely management cannot be overemphasized. Treatment of CP requires rapid diagnosis and angiographic classification. To avoid this serious and potentially lethal complication, caution must be exercised while advancing guide wires and during dilatation of the coronary lesion either before stent, during, or after stent implantation. Immediate sealing of the ruptured coronary vessel employing stent-grafts in addition to reversal of anticoagulation can defer a potentially lethal complication. Use of stent-grafts in large proximal perforations does not require any additional skill for an experienced interventional cardiologist. Nevertheless, distal coronary artery perforations may necessitate use of various embolic materials that an interventional cardiologist should be familiar with.
